# Supplementary material for: Integrating co-design into formative research for a SBCC entry-point platform for nutrition-sensitive social protection programs in low -and middle-income country settings
Source: PLoS One. 2025 Sep 15;20(9):e0310288. doi: 10.1371/journal.pone.0310288 (PMC12435663; doi:10.1371/journal.pone.0310288)
Supplement: Attachment — Submitted filename: PONE-D-24-36509.pdf [file pone.0310288.s004.pdf]

The Conceptual and Theoretical Framework

This study could be improved by providing a sound theoretical framework as the basis for the analysis. The study does not explicitly link the co-design with other established theories of behavioral change, such as health belief and TPB or social learning theory. The study could integrate a theoretical foundation to support SBCC interventions.

The assumption that co-design leads to better participation, but does not explicitly engage with limitations such as the issue of power dynamics in participatory research. Could you include examples of similar interventions on co-design that have been successful?

2. Methodological Weaknesses

The sample size of 30 SSI and 12 FDG could not have been enough to support the generalized findings of other LMICs

This should be acknowledged in the limitations.

The study does not explicitly explain how the participants were recruited or the sampling technique adopted.

It was also claimed that saturation was achieved, but no evidence was provided. Could it be coding framework stability or redundancy in responses?

What inter-coder reliability measure was employed?

Analysis and results

The thematic analysis is highly subjective, and it lacks quantitative validation. You could include basic frequency statistics to strengthen the themes.

The practical challenges, such as scalability and sustainability, were not addressed adequately despite the theoretical benefits of co-design.

The study used redundant phrases. For instance, "The needs assessments and integrated co-design sessions highlighted the benefits of close consultation with program beneficiaries in the design of culturally appropriate SBCC interventions to support nutrition-sensitive social protection programs". This should be more concise.

Format the thematic analysis coding table properly

Discussion and Policy Recommendation

The study could be improved by being specific with the policy recommendation. SDGs were mentioned, but it does not explicitly detail how policymakers can effectively implement the findings.

Reference

The reference is poorly formatted.
