# Supplementary material for: Integrating co-design into formative research for a SBCC entry-point platform for nutrition-sensitive social protection programs in low -and middle-income country settings
Source: PLoS One. 2025 Sep 15;20(9):e0310288. doi: 10.1371/journal.pone.0310288 (PMC12435663; doi:10.1371/journal.pone.0310288)
Supplement: Attachment — Submitted filename: Peer Review- SBCC.docx [file pone.0310288.s003.docx]

|  | Peer Reviewer – Luqman Abass |
| --- | --- |
| Manuscript Number: | PONE-D-24-36509 |
| Article Type | Research Article |
| Full Title: | Integrating co-design into formative research for a SBCC entry-point platform for nutrition-sensitive social protection programs in low -and middle-income country settings |
| Short Title: | SBCC entry-point platform for nutrition-sensitive social protection programs |
| Originality and Novelty | The study presents original research integrating co-design approaches into formative research for Social and Behavior Change Communication (SBCC) in nutrition-sensitive social protection programs. This integration of co-design is a novel approach in this context, particularly for low- and middle-income countries like Bangladesh and Pakistan |
| Methodology and Technical Standards | The research methodology is described in detail and appears to be of high technical standard:   - The study employs a mixed-methods approach, combining a systematic review with qualitative research methods (semi-structured interviews and focus group discussions). - The use of the COREQ checklist ensures best practice in qualitative research design, analysis, and reporting. - The integration of co-design sessions within the focus group discussions is an innovative approach to enhance participant engagement and ownership. - Data analysis utilizes Grounded Theory and thematic analysis, supported by NVIVO 14 software, which are appropriate for this type of qualitative research. |
| Data Presentation and Support for Conclusions | The manuscript provides a comprehensive description of the study design and methodology. However, as this appears to be a methods paper, the results and conclusions are not fully presented in the provided excerpt. A full assessment of data presentation and support for conclusions would require the complete manuscript. |
| Clarity and Language | The article is written in standard English and generally presented in an intelligible fashion. Its structure follows a typical scientific manuscript format, with clear sections for background, methods, and (presumably) results and discussion. |
| Ethical Standards | The study adheres to ethical standards:   - Ethics approval was obtained from appropriate institutional review boards in both Bangladesh and Pakistan. - Informed consent was requested from all participants |
| Reporting Guidelines and Data Availability | The study adheres to appropriate reporting guidelines:   - The use of the 32-item COREQ checklist ensures compliance with qualitative research reporting standards.   However, the excerpt does not provide information about data availability. This should be addressed in the full manuscript. |
| Recommendations | 1. Results and Discussion: Ensure that the full manuscript includes a comprehensive presentation of results and a thorough discussion of the findings.  2. Data Availability: Include a statement about data availability in the full manuscript.  3. Implications: Elaborate on the practical implications of integrating co-design approaches in SBCC for nutrition-sensitive social protection programs.  4. Limitations: Discuss any limitations of the study and how they were addressed or might affect the interpretation of results.  5. Future Research: Suggest directions for future research based on the findings of this study. |
